# Novel *HAX1* Gene Mutation in a Vietnamese Boy with Severe Congenital Neutropenia

**DOI:** 10.1155/2018/2798621

**Published:** 2018-11-27

**Authors:** Tham Thi Tran, Quang Van Vu, Taizo Wada, Akihiro Yachie, Huong Le Thi Minh, Sang Ngoc Nguyen

**Affiliations:** ^1^Department of Pediatrics, Haiphong University of Medicine and Pharmacy, Haiphong, Vietnam; ^2^Department of Pediatrics, Institute of Medical, Pharmaceutical and Health Science, Kanazawa University, Kanazawa, Japan; ^3^National Hospital of Pediatrics, Hanoi, Vietnam

## Abstract

Severe congenital neutropenia (SCN) is a rare disease that involves a heterogeneous group of hereditary diseases. Mutations in the *HAX1* gene can cause an autosomal recessive form of SCN-characterized low blood neutrophil count from birth, increased susceptibility to recurrent and life-threatening infections, and preleukemia predisposition. A 7-year-old boy was admitted due to life-threatening infections, mental retardation, and severe neutropenia. He had early-onset bacterial infections, and his serial complete blood count showed persistent severe neutropenia. One older sister and one older brother of the patient died at the age of 6 months and 5 months, respectively, because of severe infection. Bone marrow analysis revealed a maturation arrest at the promyelocyte/myelocyte stage with few mature neutrophils. In direct DNA sequencing analysis, we found a novel homozygous frameshift mutation (c.423_424insG, p.Gly143fs) in the *HAX1* gene, confirming the diagnosis of SCN. The patient was successfully treated with granulocyte colony-stimulating factor (G-CSF) and antibiotics. A child with early-onset recurrent infections and neutropenia should be considered to be affected with SCN. Genetic analysis is useful to confirm diagnosis. Timely diagnosis and suitable treatment with G-CSF and antibiotics are important to prevent further complication.

## 1. Introduction

Severe congenital neutropenia (SCN) is a rare disease that involves a heterogeneous group of inherited disorders. It is characterized by persistent severe neutropenia from birth, increased susceptibility to severe bacterial infections, and a preleukemic predisposition [1–3]. SCN presents several genetic inheritance states including autosomal dominant, autosomal recessive, and X-linked sporadic form, which could show association with several distinct genes [2, 4, 5]. Recent reports show that homozygous mutations in the *HAX1* gene are responsible for an autosomal recessive form of SCN, in about one-third of SCN patients [6]. *HAX1* is located mainly in the mitochondria and controls the integrity of the internal mitochondrial membrane potential and protects the myeloid cells from apoptosis [7]. Clinical signs of SCN are often overlapped with infectious diseases, sometimes causing delayed or missed diagnosis [3]. Herein, we report an SCN patient with a novel homozygous frameshift mutation in the *HAX1* gene in an attempt to improve the diagnosis and management of SCN.

## 2. Case Presentation

**Figure 1:**
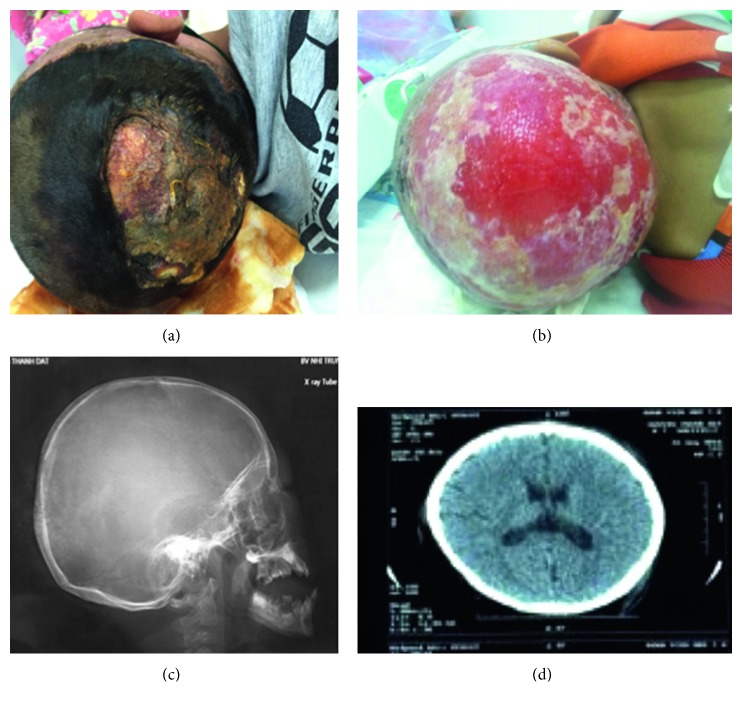
Some lesions of the patient's scalp and his skull. (a) Scalp infection. (b) Scalp peeling. (c, d) Subcutaneous emphysema of the scalp, neither brain injury nor skull fractures.

**Figure 2:**
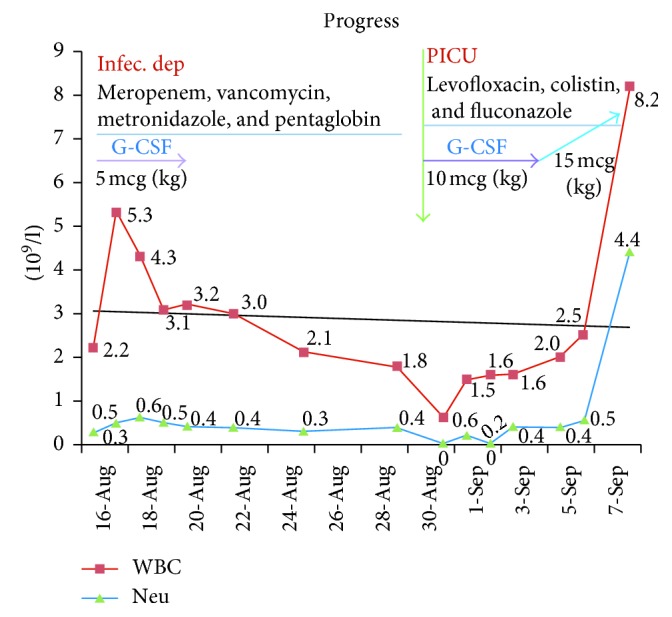
Absolute neutrophil count and total white blood cell count of the patient during treatment with G-CSF and antibiotics.

A 7-year-old boy was admitted to our hospital with a 4-day history of high fever and scalp swelling with ulcers. Physical examination revealed consciousness (Glasgow Coma Scale/core was 15), pus formation, and fistula with purulent discharge on the scalp, scalp peeling, face swelling, and poor eating (Figure 1). Laboratory findings exhibited severe neutropenia (white blood cells, 2.39 × 10^9^/l; neutrophils, 0.25 × 10^9^/l; and lymphocytes, 2.1 × 10^9^/l) and increased acute-phase reactants (erythrocyte sedimentation rate 101 mm/hour and C-reactive protein 272 mg/dl). Pus culture exhibited *Enterococcus faecalis* and *Escherichia coli*. Blood culture and urine culture were negative. The chest X-ray and urinalysis results were normal. Cerebrospinal fluid (CSF) analysis was normal. Serum titers of IgG, IgM, IgA, and IgE and percentage of CD4^+^ and CD8^+^ T cells were normal. Tests of HIV, HBV, HCV, EBV, and CMV were negative. Bone marrow analysis revealed a maturation arrest at the promyelocyte/myelocyte stage with few mature neutrophils; there was no evidence of malignant involvement in the bone marrow. Computed tomography scan of the head and skull showed subcutaneous emphysema of the scalp, neither brain injury nor skull fractures (Figure 1). Necrotizing fasciitis of the scalp and septicaemia were diagnosed. The patient was treated with pentaglobin (0.5 g/kg) and the combination of three antibiotics: vancomycin, meropenem, and metronidazole, respectively. To maintain the neutrophil count, granulocyte colony-stimulating factor (G-CSF) was administered from 5 to 10 *µ*g/kg/day and 15 *µ*g/kg/day, respectively (Figure 2). The patient was discharged from our hospital after 46 days of treatment. Now, he is well under regular G-CSF therapy.

Due to severe neutropenia and infections, we analyzed the medical history, family history, and medical records of the patient carefully. The patient is the third child in his family. He has two healthy younger sisters, one older sister who died at the age of 6 months because of meningitis, and one older brother who died at the age of 5 months because of severe pneumonia. No consanguinity was reported among parents, but their origins are from the same commune. From 7 months of age, the patient had recurrent severe infections such as cutaneous abscesses, otitis media, and respiratory infections, which were treated with appropriate antibiotics. In addition, he had neutropenia many times and mental retardation such as developmental delay, dysarthria, and linguistic immaturity.

Considering his past history, family history, and physical examination, SCN associated with *ELANE* or *HAX1* abnormality was suspected. The *ELANE* gene was analyzed by direct DNA sequencing analysis firstly but the mutation was not found. Due to mental retardation of the patient, the *HAX1* gene was analyzed next. In exon 3 of the *HAX1* gene, we found a homozygous frameshift mutation (c.423_424insG, p.Gly143fs). This is a novel mutation.

## 3. Discussion

**Figure 3:**
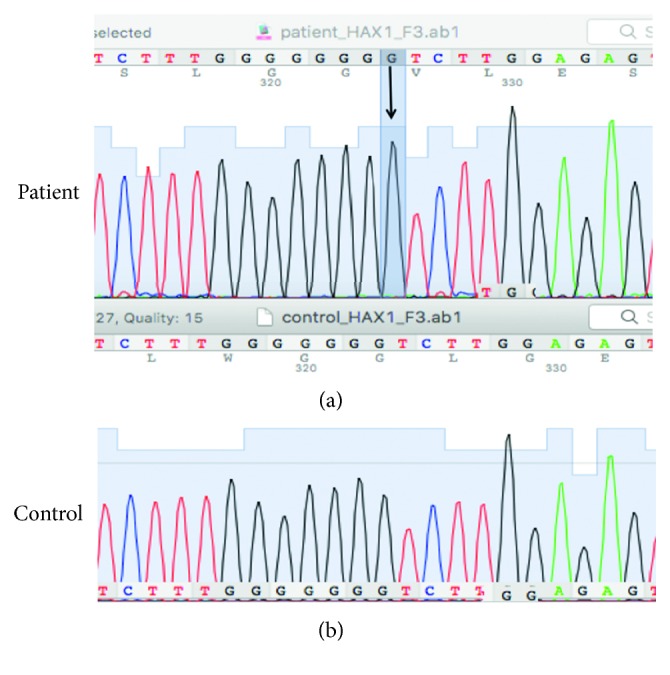
A novel homozygous frameshift mutation (c.423_424insG, p.Gly143fs) found in the *HAX1* gene (exon 3).

SCN is a rare primary immunodeficiency syndrome [8] and is associated with multiple genes including the *ELANE*, *HAX1*, *WAS*, *GFI1*, and *G6PC3* genes [7]. There are two major subtypes of SCN: autosomal dominant subtypes such as neutrophil elastase mutations (about 60% of patients) and autosomal recessive subtypes such as *HAX1* mutation (about 30% of patients), both of which share the same clinical and morphological phenotype [9]. SCN is diagnosed when ANC is less than 0.5 × 10^9^/l for at least 3 months; SCN patients suffer from recurrent life-threatening infections. The boy we report here showed typical SCN manifestations, including chronic severe neutropenia and recurrent bacterial infections. However, the diagnosis was missed and postponed to 7 years of age. This issue may be due to an inadequate knowledge about this very rare disease and because infectious diseases are popular in Vietnamese pediatric population [3, 10]. Therefore, it is important to stress this condition among health care professionals. After carefully analyzing clinical courses and bone marrow aspiration test of the patient, we excluded autoimmune neutropenia (AIN). In contrast to SCN patients, AIN patients often have mild phenotypes with minor intercurrent infections despite severe neutropenia. Because the patient had severe phenotypes with life-threatening infections, chronic severe neutropenia, and reduced granulocyte cell line on the bone marrow aspirate, SCN was diagnosed. After receiving G-CSF (from 5 to 15 *µ*g/kg/24h), his neutrophil counts increased dramatically (Figure 2). To confirm SCN diagnosis, we analyzed the *ELANE* gene mutation firstly because it is the most common gene alteration in SCN; however, no mutation was found. Because the patient has had mental retardation, the *HAX1* gene was selected for analysis next. In exon 3, we found a novel homozygous frameshift mutation (c.423_424insG, p.Gly143fs), resulting in a completely different translation from the original. To our knowledge, this is the first *HAX1* mutation report from Vietnamese people. The *HAX1* gene provides instructions for producing a protein called HS-1, which is associated with the X-1 protein (HAX-1). This protein is involved in the modulation of apoptosis, in which cells destroy themselves when damaged or no longer necessary. HAX-1 protein is found mainly in the mitochondria, the centers of energy production in cells [11]. *HAX1* gene mutations that cause SCN lead to the production of nonfunctional HAX-1 protein. The lack of functional HAX-1 protein interrupts the regulation of apoptosis, leading to premature death of neutrophils. A lack of neutrophils causes recurrent infections, inflammatory episodes, and other immune problems in patients with SCN [11, 12]. Our patient had many superficial abscesses on his scalp, which caused scalp peeling (Figure 1). This rare condition in SCN can be explained by severe infections and sheath weakness, a connective tissue disorder caused by an HAX1 deficiency [1]. However, more studies are necessary to clarify the function of the HAX1 molecule in this regard [1]. Delayed mental development in this patient was a clue to help us decide on the *HAX1* gene analysis after excluding *ELANE* gene mutation. A novel homozygous frameshift mutation (c.423_424insG, p.Gly143fs) was found on exon 3 of the *HAX1* gene (Figure 3). To date, there are few reports about *HAX1* gene mutations in SCN patients, most of which are of Middle East descent [5, 7, 13]. The *HAX1* gene includes transcript variants 1 and 2. While neurological disorders are not present in mutations associated with transcript variant 1, mutations affecting both transcript variants 1 and 2 cause SCN and neurological abnormalities such as mental retardation, epilepsy, and developmental delay [4, 7, 13]. The *HAX1* mutation of our patient is found in exon 3 affecting transcript variants 1 and 2; the patient presents mental retardation.

In summary, we report a Vietnamese boy with SCN caused by a novel *HAX1* gene mutation. Every child with early-onset recurrent infections and neutropenia should be considered to have SCN. Genetic analysis is useful to confirm the diagnosis. Timely diagnosis and suitable treatment with G-CSF and antibiotics are important to prevent further complication.

## Data Availability

The data used to support the findings of this study are included within the article.

## Abbreviations

G-CSF: Granulocyte colony-stimulating factor
HAX1: HS1-associated protein X1
ELANE: ELA2 elastase, neutrophil expressed
SCN: Severe congenital neutropenia.

## References

[1] Eghbali A., Eshghi P., Malek F., Abdollahpour H., Rezaei N. (2010). HAX1 mutation in an infant with severe congenital neutropenia. *Turkish Journal of Pediatrics*.

[2] Xue S.-L., Li J.-L., Zou J.-Y., Su J., Chen S.-N., Wu D.-P. (2012). A novel compound heterozygous HAX1 mutation in a Chinese patient with severe congenital neutropenia and chronic myelomonocytic leukemia transformation but without neurodevelopmental abnormalities. *Haematologica*.

[3] Vu Q. V., Wada T., Tran T. T. (2015). Severe congenital neutropenia caused by the ELANE gene mutation in a Vietnamese boy with misdiagnosis of tuberculosis and autoimmune neutropenia: a case report. *BMC Hematology*.

[4] Faiyaz-Ul-Haque M., Al-Jefri A., Al-Dayel F. (2010). A novel HAX1 gene mutation in severe congenital neutropenia (SCN) associated with neurological manifestations. *European Journal of Pediatrics*.

[5] Smith B. N., Ancliff P. J., Pizzey A., Khwaja A., Linch D. C., Gale R. E. Homozygous HAX1 mutations in severe congenital neutropenia patients with sporadic disease: a novel mutation in two unrelated British kindreds. *British Journal of Haematology*.

[6] Klein C., Grudzien M., Appaswamy G. (2007). HAX1 deficiency causes autosomal recessive severe congenital neutropenia (Kostmann disease). *Nature Genetics*.

[7] Aydogmus C., Cipe F., Tas M. HAX-1 deficiency: characteristics of five cases including an asymptomatic patient. *Asian Pacific Journal of Allergy and Immunology*.

[8] Germeshausen M., Grudzien M., Zeidler C. (2008). Novel HAX1 mutations in patients with severe congenital neutropenia reveal isoform-dependent genotype-phenotype associations. *Blood*.

[9] Zeidler C., Germeshausen M., Klein C., Welte K. (2009). Clinical implications of ELA2-, HAX1-, and G-CSF-receptor (CSF3R) mutations in severe congenital neutropenia. *British Journal of Haematology*.

[10] Vu Q. V., Wada T., Le H. T. M. (2014). Clinical and mutational features of Vietnamese children with X-linked agammaglobulinemia. *BMC Pediatrics*.

[11] HAX1 gene. Genetics Home Reference. https://ghr.nlm.nih.gov/gene/HAX1

[12] Klein C. (2017). Kostmann’s disease and HCLS1-associated protein X-1 (HAX1). *Journal of Clinical Immunology*.

[13] Lanciotti M., Indaco S., Bonanomi S. (2010). Novel HAX1 gene mutations associated to neurodevelopment abnormalities in two Italian patients with severe congenital neutropenia. *Haematologica*.

